# Dietary Stevia Residue Extract Supplementation Improves Antioxidant Capacity and Intestinal Microbial Composition of Weaned Piglets

**DOI:** 10.3390/antiox11102016

**Published:** 2022-10-12

**Authors:** Shuai Liu, Yunxia Xiong, Shuting Cao, Xiaolu Wen, Hao Xiao, Yajing Li, Lei Chi, Dongsheng He, Zongyong Jiang, Li Wang

**Affiliations:** 1 State Key Laboratory of Livestock and Poultry Breeding, Ministry of Agriculture Key Laboratory of Animal Nutrition and Feed Science in South China, Guangdong Key Laboratory of Animal Breeding and Nutrition, Maoming Branch, Guangdong Laboratory for Lingnan Modern Agriculture, Institute of Animal Science, Guangdong Academy of Agricultural Sciences, Guangzhou 510640, China; 2 Zhucheng Haotian Pharmaceutical Co., Ltd., Zhucheng 262218, China

**Keywords:** stevia residue extract, antioxidant, gut microbiota, weaned piglets

## Abstract

This study aimed to investigate the effects of diet supplementation with stevia residue extract (SRE) on growth performance, intestinal health, and antioxidant capacity of weaned piglets. A total of 144 weaned piglets (body weight 6.8 ± 0.5 kg) were randomly selected and allocated into four treatment groups with six replicates of six pigs/pen. The treatments consisted of a basal diet without SRE or basal diet supplemented with 100, 200, or 400 mg/kg SRE. The results showed that the addition of 200 mg/kg SRE to the diet significantly reduced (*p* < 0.05) the diarrhea rate of piglets compared with the control group. The supplementation of 400 mg/kg SRE in the diet significantly reduced the piglets’ serum MDA content and significantly increased (*p* < 0.05) the T-AOC, T-SOD, and GSH-PX activity in the serum. The dietary supplementation with 400 mg/kg SRE significantly increased (*p* < 0.05) the CAT and GSH-PX activity in the liver. Moreover, the supplementation of 400 mg/kg SRE in the diet significantly increased (*p* < 0.05) the relative abundance of Prevotellaceae (genus) and Roseburia (genus) beneficial bacteria compared to the control group. Spearman’s correlation analysis showed that Prevotella (genus) abundance was positively correlated with liver GSH-PX activity and acetic acid content of colon contents. In conclusion, the supplementation of 400 mg/kg SRE to the diet can improve piglet health by regulating antioxidant reduction homeostasis, which may also be associated with an increase in the relative numbers of potentially beneficial bacteria.

## 1. Introduction

Under natural conditions, piglets are weaned after 17 weeks of age, but in the commercial farming model, the weaning time of piglets has been advanced to 21–28 days of age [1,2]. Early weaning disrupts the oxidative balance of the piglets and leads to oxidative damage [3]. This causes injury to the intestinal barrier which can lead to diarrhea and even death in the piglets [4]. Therefore, it is important to improve the antioxidant capacity of weaned piglets to reduce oxidative stress and diarrhea caused by weaning.

Stevia rebaudiana is a branched, bushy shrub native to the Amambay region in northeast Paraguay, and it is now widely cultivated in Asia, Europe, and North America [5,6]. Steviol glycosides are the main sweet component in stevia rebaudiana and are 300 times sweeter than sucrose [7]. Stevia rebaudiana is popular in the market because of its sweetness and low calorie content [8]. China is the world’s largest cultivator of stevia rebaudiana and producer of steviol glycosides, and production is increasing year by year [9]. However, the widespread cultivation of stevia rebaudiana and the extraction of steviol glycosides creates a potential danger to the environment. Stevia residues are usually disposed of in landfills and incinerators, which results in environmental pollution and wasted resources [10,11]. Some studies have found that most of the bioactive substances in stevia, such as flavonoids, phenolic compounds, and phytosterols, remain in the stevia residue [9,12]. Some studies have found that the main polyphenols in stevia residue are mainly chlorogenic acid and chlorogenic-acid-like substances, which have strong antioxidant activity [13,14,15]. In early studies, it was found that supplementation of chlorogenic acid in weaned piglets’ diets reduced intestinal inflammation and increased antioxidant capacity, which in turn improved the barrier function and diarrhea rate in piglets [16,17]. In addition, it has been shown that feeding diets with extracts of stevia residue improves meat quality and increases the antioxidant capacity of finishing pigs [11]. It was found that stevia residue extract has the potential to improve hyperuricemia in mice [18]. Another study showed that supplementation with SRE in the diets of mice with disorders of glucolipid metabolism was associated with significant improvements in antioxidant status and the regulation of lipid metabolism [19]. In addition, SRE was also found to attenuate D-galactose-induced oxidative damage in mice by increasing superoxide dismutase, catalase, and glutathione peroxidase enzyme activities in serum and liver [20]. However, few studies have been performed on SRE in piglets.

Therefore this study aimed to detect the effects of supplementing SRE in the diet on the growth performance, intestinal health, antioxidant capacity, intestinal microflora, and short-chain fatty acid content in the colon in weaned piglets.

## 2. Materials and Methods

All animal procedures used in the present study were approved by the Animal Care and Use Committee of Guangdong Academy of Agricultural Sciences and followed the Guidelines for the Care and Use of Animals for Research and Teaching (Authorization number GAASIAS-2016-017).

### 2.1. Experimental Design and Sample Collection

The SRE used in this study was provided by Zhucheng Haotian Pharmaceutical Co., Ltd. (Zhucheng, China). A total of 108 21-day-old weaned piglets (Duroc × Landrace × Yorkshire) with an average initial body weight of 6.80 ± 0.05 kg were randomly allocated to four treatments in a randomized complete block design based on initial body weight and sex. There were six replicates per treatment and six pigs per replicate (three male and three female). The treatments consisted of a basal diet without SRE or basal diet supplemented with 100, 200, or 400 mg/kg SRE. The whole trial lasted for 42 days. The basal diets were formulated to meet or exceed the NRC (2012) guidelines according to body weight (Table 1). The feed intake and the body weight were recorded on days 28 and 42 of the experiment to calculate average daily gain (ADG, total weight gain/number of test days), average daily feed intake (ADFI, total feed intake/(number of test days × number of piglets)), and the ratio of feed to gain (F:G, ADFI/ADG). During the experiment, each group of animals was given ad libitum access to water and food. Feed intake and diarrhea rates (total number of diarrhea events/(number of piglets × number of test days) × 100%) were accurately recorded during the experiment, and pigs were weighed and blood collected after 12 h of fasting on days 29 and 43. At the end of the experiment, one pig from each replicate was selected for sample collection. Blood and liver were collected for antioxidant capacity. The digest from the duodenum, jejunum, ileum, and colon was carefully collected and snap frozen in liquid nitrogen. Tissue samples from the proximal duodenum, middle jejunum, and distal ileum were dissected, and the samples were fixed with 4% paraformaldehyde for subsequent morphological analysis.

### 2.2. Antioxidative Capacity and Digestive Enzyme Activity Evaluation

Total antioxidant capacity (T-AOC) was determined using the ABTS method (Item No. A015-2-1), malondialdehyde (MDA) content was determined using the TBA method (Item No. A003-2), catalase (CAT) activity was determined using the ammonium molybdate method (No. A007-1-1), total superoxide dismutase (T-SOD) activity was determined using the hydroxylamine method (No. A001-1), glutathione peroxidase (GSH-Px) activity was determined using the dithiodinitrobenzoic acid method (Item No. A005), trypsin activity (Item No. A080-2) was determined using the UV colorimetric method, α-amylase (AMS) was determined using the starch–iodine colorimetric method (No. C016-1-1), and lipase (LPS) activity was determined using the colorimetric method (No. A054-1-1). The kits were purchased from Nanjing Jiancheng Institute of Biological Engineering, and the samples of each intestinal segment were homogenized and evaluated according to the instructions of the kits. Briefly, the samples were homogenized with the sample dilution at a ratio of 1:10. The homogenate was centrifuged at 4500 rpm for 10 min at 4 °C. The supernatant was taken for the enzymatic assay. The protein concentrations of the sample were determined with a BCA kit (Thermo Fisher, Waltham, MA, USA), and the final results were expressed per mg protein.

### 2.3. Intestinal Histopathological Examination

The intestinal morphology was examined according to the method of Liu et al. [21]. Briefly, after being fixed and embedded in paraffin, intestinal segments were cut into 5 μm sections. The sections were then dewaxed with xylene, hydrated with alcohol, and stained with hematoxylin and eosin (H&E). Images were obtained using a fluorescent orthochromatic microscope (Haier, Qingdao, China). Ten bright fields were randomly selected for each section. The villus height and crypt depth were measured using Pannoramtic Viewer v1.15.3 software (3DHISTECH Ltd., Budapest, Hungary).

### 2.4. Short-Chain Fatty Acid Measurement

A sample of 0.5 g of colon contents was taken and fully dissolved in 1.5 mL of water. The suspension was put into a centrifuge and centrifuged at 5000 rpm for 30 min. Next, 1 mL of supernatant was removed in a 2 mL PE tube and 200 μL of 42 mmol/L crotonic acid and 200 μL of 10% metaphosphoric acid solution was added and mixed thoroughly. The PE tube was put into the refrigerator overnight at 4 °C and then centrifuged at 10,000 rpm for 10 min at 4 °C. The supernatant was mixed with ether in equal proportions and left to extract for 5 min. The ether layer was aspirated with a disposable syringe, filtered through a 0.22 μm organic membrane, and injected into a brown injection bottle.

Short-chain fatty acids (acetic, propionic, butyric, valeric, isobutyric, and isovaleric acids, with crotonic acid as an internal standard) were measured using a gas chromatograph coupled with a mass spectrometer detector (7890A and 5975C inert XL EI/CI mass spectrometric detector, Agilent Technologies, Santa Clara, CA, USA). The column flow rate was set to 0.8 mL/min and the purge flow rate was 3.0 mL/min. The hydrogen flow rate was set to 30 mL/min, the tail blow flow rate was 25 mL/min, and the air flow rate was 400 mL/min. The inlet temperature was set to 250 °C and the detector temperature was maintained at 280 °C. The ramp-up program was set to ramp up from the initial temperature of 60 °C to 220 °C at a rate of 20 °C/min and hold for 4 min, for a total run time of 12 min. The injection volume was 1 μL and the split ratio was 50:1. The injection needle was cleaned with acetone.

### 2.5. DNA Extraction and 16S rRNA Amplification

In this experiment, 16S rRNA sequencing was applied to analyze the effect of Stevia rebaudiana extract on the colonic content flora of weaned piglets. Total genomic DNA from colon contents was extracted using the OMEGA Soil DNA Kit (cat: D5625-01; Omega Bio-Tek, Norcross, GA, USA) according to the manufacturer’s instructions and used for further analysis. The quantity and quality of total DNA extracted were assessed using a NanoDrop ND-1000 spectrophotometer (Thermo Fisher Scientific, Waltham, MA, USA) and agarose gel electrophoresis. The V3-V4 region of the bacterial 16S rRNA gene was amplified using polymerase chain reaction (PCR) amplification (CFX System, Bio-Rad, Hercules, CA, USA) with forward primer 338F (5′-ACTCCGGGAGGCAGCA-3′), reverse primer 806R (5′-TCGGACTACHVGGCAGCA-3′), and reverse primer 806R (5′-TCGGACTACHVGTWTCTAAT-3′). The PCR components contained 5 μL buffer (5×), 5 μL GC buffer (5×), 0.25 μL Fast pfu DNA Polymerase (5 U/μL), 2 μL dNTPs (2.5 mM), 109 1 μL forward and reverse primer (10 μM), 1 μL DNA template, and 8.75 μL ddH_2_O. PCR amplicons were purified with Vazyme VAHTSTM DNA cleaning beads (Vazyme, Nanjing, China) and quantified using the Quant-iT PicoGreen dsDNA detection kit (Invitrogen, Carlsbad, CA, USA). After a single quantification step, amplicons were pooled into aliquots and paired-end 2 × 250 bp sequencing was performed using the Illumina MiSeq platform and MiSeq Reagent Kit v3 at Shanghai Personal Biotechnology Co., Ltd. (Shanghai, China).

### 2.6. Bioinformatics Analysis

Bioinformatics analysis was performed using QIIME2 2019.4 with slight modifications. Briefly, raw sequence data were demultiplexed using the demultiplexing plugin, followed by primer cleavage using the cutadapt plugin. The sequences were then quality filtered, denoised, merged, and chimeras removed using the DADA2 plugin. Non-singleton amplicon sequence variants (ASVs) were aligned to mafft and used to construct the phylogeny of fasttree2. Diversity inserts were used to assess α-diversity metrics (Chao1, observed-species, Shannon, Simpson) and β-diversity metrics (weighted UniFrac heterogeneity). Taxonomy was assigned to ASVs using the classify-sklearn naïve Bayes taxonomy classifier in feature-classifier plugin against the Greengenes 13 Database. Sequence data analysis was performed mainly using QIIME2 and the R package (v3.2.0). Alpha diversity indices at the ASV level, such as Chao1, observed-species, Shannon, and Simpson indexes, were calculated using the ASV table in QIIME2 and visualized as box plots. Beta diversity analysis was performed using a weighted UniFrac distance metric to investigate structural variation in microbial communities between samples and visualized by principal coordinate analysis (PCoA) and non-metric multidimensional scaling (NMDS). LDA effect size (LEfSe) analysis and RandomForest analysis were generated or calculated by personal genescloud (https://www.genescloud.cn, accessed on 11 August 2022).

### 2.7. Statistical Analysis

The trial data in this study were analyzed using the IBM SPSS Statistics V25.0 software package (IBM Corp., Armonk, NY, USA). Significance was evaluated by one-way analysis of variance (ANOVA) for production performance, digestive enzyme activity, antioxidant capacity, intestinal morphology, and short-chain fatty acids. Homogeneity of variance was tested with Levene’s test. ANOVA followed by Tukey’s multiple-comparison test was performed for multiple comparisons. In the case of unequal variances, Kruskal–Wallis test was followed by the Dunn test, and false discovery rate (FDR) values were estimated using the Bonferroni method to control for multiple testing. The relative abundances of microbes were compared by Kruskal–Wallis test followed by the Dunn test, and false discovery rate (FDR) values were estimated using the Bonferroni method to control for multiple testing. Correlation analysis was measured using Spearman’s correlation coefficients. *p* < 0.05 was considered a significant difference, and 0.05 ≤ *p* < 0.10 was considered a significant tendency.

## 3. Results

### 3.1. Components of SRE

The components of SRE other than moisture, ash, crude protein, and crude fat were determined using high-performance liquid chromatography. As in our previous reports, the main components of SRE are shown in Table 2 [11]. The most abundant substance in SRE was β-glucan at 19.50%. The second most abundant substance was isochlorogenic acid A at 16.66%. The third most abundant substance was chlorogenic acid at 13.69%. In addition, chlorogenic acid and chlorogenic acid analogues (e.g., isochlorogenic acid, neochlorogenic acid, and quinic acid) accounted for more than 40% of the content.

### 3.2. Performance of Weaned Piglets

As shown in Table 3, compared with the control group, the supplementation of SRE in the diet had no significant effect on the average daily weight gain, average daily feed intake, and feed-to-gain ratio of piglets (*p* > 0.05). However, compared with the control group, the diarrhea rate of piglets was significantly decreased (*p* < 0.05) in the 100 mg/kg SRE supplementation group and 200 mg/kg SRE supplementation group during days 29–42. In addition, the piglets fed diets supplemented with 200 mg/kg SRE showed a significantly reduced (*p* < 0.05) diarrhea rate during days 1–42 compared to the control group.

### 3.3. Serum and Liver Antioxidant Capacity

The serum antioxidant capacity indexes at days 28 and 42 of the trial are shown in Table 4. At day 28 of the trial, compared with the control group, the MDA contents of piglets’ sera were significantly reduced (*p* < 0.05) in those fed the diet supplemented with 100 mg/kg and 200 mg/kg SRE group. The MDA contents of those fed the diet with 400 mg/kg SRE were not significantly different (*p* > 0.05) from those of the other three groups. T-SOD activity was significantly increased (*p* < 0.05) in the group fed diets supplemented with 200 mg/kg SRE and in the group fed diets supplemented with 100 mg/kg SRE compared to the control group. However, there was no significant change (*p* > 0.05) in CAT activity in either group. GSH-PX activity was significantly increased (*p* < 0.05) in the group fed diets supplemented with 400 mg/kg SRE compared to the control group. Finally, the results of serum T-AOC content in piglets showed that those fed diets with 200 and 400 mg/kg SRE groups had significantly increased levels (*p* < 0.05) compared to the control group. At day 42 of the trial, compared with the control group, the serum MDA content of piglets fed the diet supplemented with SRE was significantly reduced (*p* < 0.05). Correspondingly, the serum T-SOD activity of piglets in all groups fed diets supplemented with SRE was significantly increased (*p* < 0.05) compared to the control group. In addition, serum CAT activity was significantly increased (*p* < 0.05) in the piglets fed diets supplemented with 100 mg/kg SRE compared to the control piglets. Compared to other groups, serum GSH-PX activity was significantly increased (*p* < 0.05) in piglets fed diets with 200 mg/kg SRE and 400 mg/kg SRE. However, the serum T-AOC results were not significantly different among the groups (*p* > 0.05).

The liver antioxidant capacity index results at day 42 are shown in Table 5. Compared to the control group, the liver MDA content of piglets fed diets supplemented with 200 mg/kg SRE was significantly reduced (*p* < 0.05). However, the liver T-SOD activity of piglets in the group fed diets supplemented with 200 mg/kg SRE was not significantly different from that of the control group (*p* > 0.05). The liver T-SOD activity of piglets fed diets supplemented with 400 mg/kg SRE was also not significantly different from that of the control group (*p* > 0.05). However, the liver T-SOD activity of piglets fed diets supplemented with 100 mg/kg SRE was significantly increased (*p* < 0.05) compared to the groups fed diets supplemented with 200 mg/kg and 400 mg/kg SRE. Compared with the other three groups, the liver CAT activity of piglets in the group fed diets supplemented with 400 mg/kg SRE was significantly increased (*p* < 0.05). In addition, the liver GSH-PX activity was also significantly increased (*p* < 0.05) in piglets fed the diet supplemented with 400 mg/kg SRE compared to the control group and the group fed diets supplemented with 200 mg/kg SRE. However, there was no significant difference in the liver T-AOC of piglets in each group (*p* > 0.05).

### 3.4. Small Intestinal Digestive Enzyme Activity and Morphology

The results for enzyme activity in duodenum, jejunum, and ileum are shown in Table 6. Compared with the control group, the trypsin activity in the duodenum of piglets fed diets supplemented with 100 mg/kg SRE showed a decreasing trend (0.05 ≤ *p* < 0.1). In addition, compared with the control group, the amylase activity in the duodenum of piglets fed diets supplemented with 400 mg/kg SRE showed a decreasing trend (0.05 ≤ *p* < 0.1), and the lipase activity in the duodenum of piglets fed diets supplemented with 200 mg/kg SRE showed a decreasing trend (0.05 ≤ *p* < 0.1). However, compared to the control group, the supplementation of SRE in the diet had no significant effect (*p* > 0.05) on digestive enzyme activity in the jejunum and ileum of piglets.

**Figure 1:**
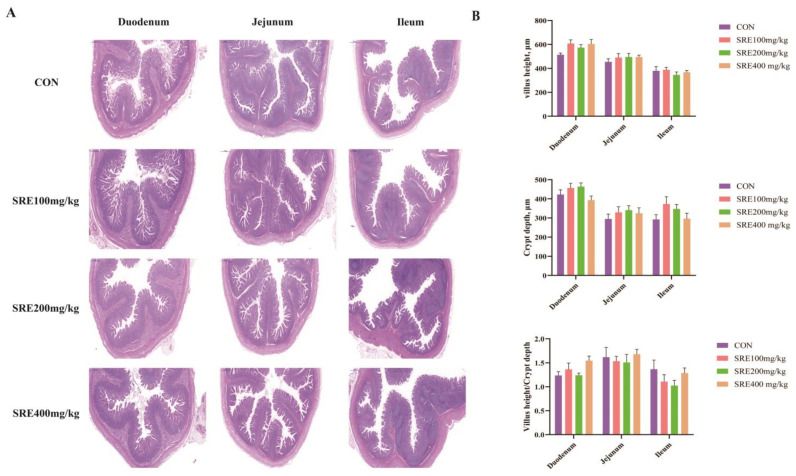
Effects of supplementation of stevia residue extract in the diet on small intestine morphology of weaned piglets on day 42 of the trial. Hematoxylin–eosin staining photos of the duodenum, jejunum, and ileum (magnification 20×) (**A**); the morphology of villus height, crypt depth, and villus height/crypt depth (**B**). Con: control group without the supplementation of stevia residue extract in the diet; SRE100 mg/kg: diets supplemented with 100 mg/kg stevia residue extract group; SRE200 mg/kg: diets supplemented with 200 mg/kg stevia residue extract group; SRE400 mg/kg: diets supplemented with 400 mg/kg stevia residue extract group. Values are means ± SEM, n = 6. Labeled means in a row with different letters differ, *p* < 0.05.

The effects of the diets supplemented with stevia residue extract on the morphology of the small intestines of piglets are shown in Figure 1. As shown in Figure 1A, the morphology of the duodenum, jejunum, and ileum was not significantly injured in any group. Figure 1B shows the small intestinal villus height, crypt depth, and villus height to crypt depth ratio. Similar to the results in Figure 1A, the small intestinal villus height, crypt depth, and villus height to crypt depth ratio were not significantly different between groups (*p* > 0.05).

### 3.5. Colon Microbes

**Figure 2:**
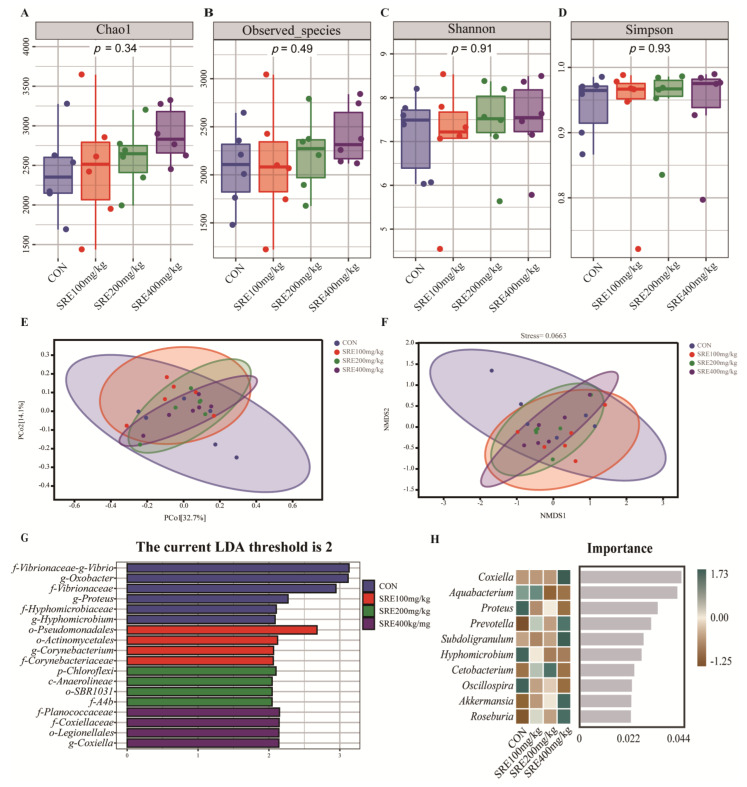
Effects of supplementation of diets with stevia residue extract on colonic microbial diversity of weaned piglets on day 42 of the trial. Comparison of alpha diversity index (Chao1 index, Good’s coverage index, Shannon index, Simpson index) among the three groups (**A**–**D**). Bray−Curtis distance-based principal coordinates analysis (**E**). Bray−Curtis distance-based nonmetric multidimensional scaling plot (**F**). The LEfSe analysis (LDA score ≥ 2) identified the biomarker bacterial species in the three groups (**G**). Top 10 marker species in each group at the genus level were analyzed using Random Forest Classifier (**H**). Con: control group without the supplementation of stevia residue extract in the diet; SRE100 mg/kg: diets supplemented with 100 mg/kg stevia residue extract group; SRE200 mg/kg: diets supplemented with 200 mg/kg stevia residue extract group; SRE400 mg/kg: diets supplemented with 400 mg/kg stevia residue extract group.

The effects of SRE supplementation in the diet on the colonic microbial diversity of piglets are shown in Figure 2. The supplementation of SRE in the diet had no significant effect (*p* > 0.05) on the Chao1 index, observed-species index, Shannon index, or Simpson index of colon microbes in piglets compared to the control group (Figure 2A–D). As shown in the PCoA plot (Figure 2E) and NMDS plot (Figure 2F), there was no significant separation between the groups. LEfSe (LDA scores > 2) was used to analyze the differential bacteria in each group. The results showed that 18 differential bacteria were identified in the four groups (Figure 2G). The top 10 marker species of each group at the genus level were analyzed using a random forest classifier (Figure 2H). It was found that Coxiella (genus), Prevotella (genus), Subdoligranulum (genus), Akkermansia (genus), and Roseburia (genus) were enriched in the intestines of piglets fed diets with 400 mg/kg SRE.

**Figure 3:**
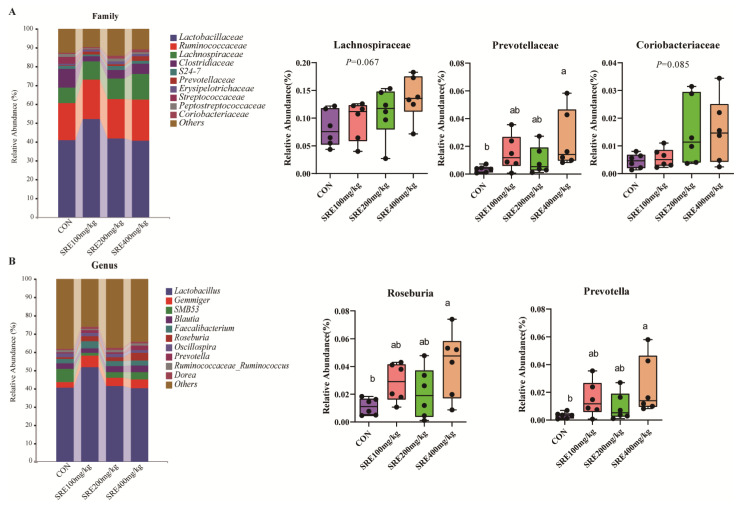
Effects of supplementation with stevia residue extract in the diet on the colonic microbial composition of weaned piglets on day 42 of the trial. The stacked bar chart on the left shows the top 10 species in relative abundance at the family level, and the box plot on the right shows the differential species among the top 10 species at the family level (**A**); the stacked bar chart on the left shows the top 10 species in relative abundance at the genus level. The box plot on the right shows the differential species among the top 10 species at the genus level (**B**). Con: control group without the supplementation of stevia residue extract in the diet; SRE100 mg/kg: diets supplemented with 100 mg/kg stevia residue extract group; SRE200 mg/kg: diets supplemented with 200 mg/kg stevia residue extract group; SRE400 mg/kg: diets supplemented with 400 mg/kg stevia residue extract group. The relative abundance of microbes was compared by Kruskal–Wallis test followed by the Dunn test, and false discovery rate (FDR) values were estimated using the Bonferroni method to control for multiple testing (n = 6). Labeled means in a row with different letters differ, *p* < 0.05.

The effect of SRE supplementation in the diet on the microbial composition of the colon is shown in Figure 3. At the family level, Lactobacillaceae (family, 40.72–52.20%), Ruminococcaceae (family 19.78–21.65%), and Lachnospiraceae (family 8.14–13.72%) were the major families (68.81–82.65%) (Figure 3A). At the family level, among the top 10 bacteria of relative abundance, there was an increased trend in the relative abundance (0.05 < *p* < 0.1) of Lachnospiraceae (family) and Coriobacteriaceae (family) in the group fed diets supplemented with 400 mg/kg SRE compared to the control group (Figure 3A). In addition, the relative abundance of colon Prevotellaceae (family) was significantly increased (*p* < 0.05) in the piglets fed diets containing 400 mg/kg SRE compared to the control group (*p* < 0.05) (Figure 3A). At the genus level, among the top 10 bacteria of relative abundance, compared to the control group, the relative abundance of Roseburia (genus) and Prevotella (genus) was significantly increased (*p* < 0.05) in the group fed diets with 400 mg/kg SRE (Figure 3B).

### 3.6. Short-Chain Fatty Acid Content of Colon Contents and Correlation Analysis of Short-Chain Fatty Acid Content with Significantly Changed Microorganisms

The results of the measurement of the short-chain fatty acid content of the colon contents are shown in Table 7. The results showed that the contents of acetic, propionic, butyric, and valeric acids in the colon contents of piglets fed diets supplemented with SRE were not significantly changed compared to the control group (*p* > 0.05). However, isobutyric acid and isovaleric acid contents of colon contents were significantly reduced in piglets supplemented with 100 mg/kg SRE in the diet compared to the control group and the 400 mg/kg SRE supplementation group (*p* < 0.05).

### 3.7. Spearman’s Correlation Analysis

**Figure 4:**
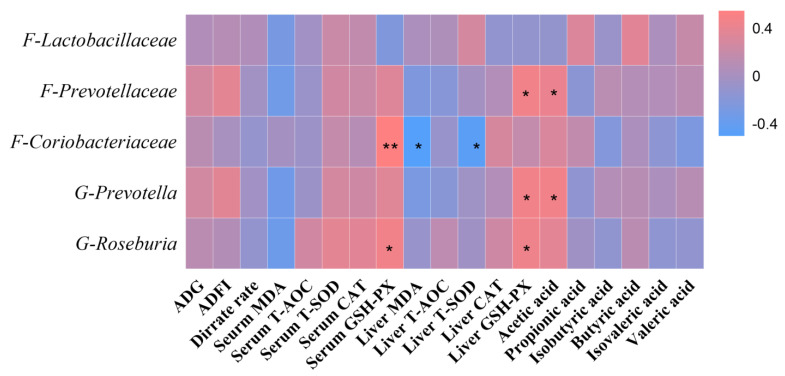
Spearman’s correlation was used to measure the correlation of colon microbes with the short-chain fatty acid content of colon contents, 1–42 day production performance, serum antioxidant capacity, and liver antioxidant capacity. ADG: average daily gain; ADFI: average daily feed intake; MDA: malondialdehyde; TAOC: malondialdehyde; T−SOD: total superoxide dismutase; CAT: catalase; GSH−Px: glutathione peroxidase. In the heatmap of the correlation coefficient, the red represents positive correlations and the blue represents negative correlations (* presented *p* < 0.05, ** presented *p* < 0.01).

The results of analysis of the significantly varying abundances of colonies with short-chain fatty acid content, 1–42 day production performance, serum antioxidant capacity, and liver antioxidant capacity using Spearman’s correlation coefficient are shown in Figure 4. The results showed that Coriobacteriaceae (family) and Roseburia (genus) had a significant positive (*p* < 0.05) correlation with serum GSH-PX on day 42 of the test. Coriobacteriaceae (family) showed a significant negative (*p* < 0.05) correlation with hepatic MDA and T-sod. Prevotellaceae (family), Roseburia (genus), and Prevotella (genus) showed a significant positive (*p* < 0.05) correlation with hepatic GSH-PX on day 42 of the test. Prevotellaceae (family), Roseburia (genus), and Prevotella (genus) were significantly and positively correlated with liver GSH-PX (*p* < 0.05). Lachnospiraceae (family), Prevotellaceae (family), Coriobacteriaceae (family), Roseburia (genus), and Prevotella (genus) were not significantly correlated with acetic acid, propionic acid, butyric acid, valeric acid, isobutyric acid, or isovaleric acid (*p* > 0.05). However, both Prevotellaceae (family) and Prevotella (genus) had a significant positive correlation with acetic acid (*p* < 0.05).

## 4. Discussion

The reasonable utilization of stevia residue can reduce environmental pollution. In previous studies, stevia and its extracts have been found to have antioxidant, anti-inflammatory, antibacterial, and glucose and lipid metabolism-modulating properties [22,23,24,25]. There are studies suggesting that the physiological functions of stevia may be related to the bioactive substances in stevia, such as chlorogenic acid, flavonoids, quercetin, and protocatechuic acid [9,26,27]. However, most steviol glycoside processing plants still discard the bioactive substances in the solid stevia residue produced during the stevia production process [9]. Some studies have found that the main polyphenol in stevia residues is chlorogenic acid [9,14,26]. In the present study, we used stevia residue extract, a brown powder with a high percentage of bioactive substances such as chlorogenic acid, chlorogenic acid, and β-glucan up to 68.48%. The physiological functions of chlorogenic acid, chlorogenic acid, and β-glucan have been widely reported in previous studies, including antioxidant, antibacterial, and immune enhancement [28,29,30]. These functions also give stevia residue extract the potential to be used as a piglet feed additive. Our previous study reported that feeding diets supplemented with stevia residue extract to finishing pigs resulted in improved production performance, meat quality, and antioxidant capacity [11]. In the conclusion, the use of SRE as a feed additive may have potential benefits for piglets. Therefore, this study is the first to report the effects of stevia residue extract on performance, intestinal function, and antioxidant capacity in weaned piglets.

In this experiment, we found that the supplementation with stevia residue extract at 100, 200, and 400 mg/kg in the diet had no significant effect on the average daily weight gain, average daily feed intake, and feed-to-weight ratio of weaned piglets. However, the results of our experiments on finishing pigs found that supplementation of 100 mg/kg of stevia residue extract in the diet improved the average daily weight gain and average daily feed intake of fattening pigs [11]. This may be related to the different degrees of digestive system development between piglets and finishing pigs. However, the addition of SRE can reduce the rate of piglet diarrhea. In a study on β-glucan (highest percentage of substances in SRE), it was found that the addition of β-glucan to the diet also had no significant effect on the growth performance of piglets [31]. Another study found that the supplementation of chlorogenic acid (the third most abundant substance in SRE) in the diet increased the average daily weight gain of piglets and reduced the feed-to-weight ratio [32]. It was found that supplementation with Eucommia ulmoides leaf extracts, which were composed mainly of polysaccharides, flavonoids, and chlorogenic acid, had no significant effect on the performance of piglets [33]. At present, the functions of different plant extracts and bioactive substances of plant origin in improving growth performance are still controversial.

It is well known that pigs are exposed to adverse stresses such as environmental changes, transportation, and feed contamination from birth to slaughter. These stresses may eventually cause oxidative stress due to the redox homeostasis in the pigs’ bodies [34]. Oxidative stress is especially evident during the weaning period of piglets, and it can even cause their death [35]. Antioxidants are often added to piglet feed during production to reduce the level of oxidative stress. Most studies in the field of SRE have only focused on in vitro studies or in vivo studies in mice. Zhao et al. [20] found that SRE scavenged various free radicals and significantly increased serum T-AOC content and T-SOD activity and decreased MDA content in oxidatively stressed mice by activating the Akt/Nrf2/HO-1 pathway. Another study by Zhao et al. [19] also showed that SRE can improve serum antioxidant status in mice. In our study, we found that the addition of SRE significantly improved the antioxidant capacity of the liver and serum in piglets. This is consistent with the results we obtained on fattening pigs [11]. A previous study found that the supplementation of chlorogenic acid in piglet diets increased serum GSH-PX activity, increased duodenal GSH-PX and CAT activity, and decreased ileal MDA levels [32]. This mainly manifested as reduced MDA content and increased T-SOD, CAT, and GSH-PX activity. Isochlorogenic acid from SRE has been reported to reduce nitric oxide production in LPS-induced inflammatory mouse mononuclear macrophages [36].Isochlorogenic acid contains multiple R-OH radicals, which can form hydrogen radicals with antioxidant activity and can remove hydroxyl radicals and superoxide anions, thereby reducing tissue oxidative damage [37]. Moreover, the antioxidant bioactivity of β-glucan has been reported in previous studies [38]. Wu et al. [39] showed that the addition of 200 mg/kg of β-glucan to the diet did not significantly change the antioxidant capacity of weaned piglets. Meanwhile, He et al. [40] found that the addition of 200 mg/kg of β-glucan to the diet improved the antioxidant capacity of finishing pigs. We also measured β-glucan at 19.5%, which may also contribute to the antioxidant potential of SRE. Thus, the effect of SRE in improving the antioxidant capacity of weaned piglets may be related to a variety of components. However, further studies are needed on the contributions of various components to antioxidant capacity and the associated mechanisms.

Weaning stress tends to lead to physiological changes in the intestinal structure and function of piglets, which are often detrimental [41]. However, in the present study, no such changes were observed in the control and test groups. It is noteworthy that we found changes in the microorganisms in the colons of piglets fed the 400 mg/kg diet. Compared to the control, at the genus level, we found that the addition of 400 mg/kg SRE to the diet significantly increased the relative abundances of Roseburia and Prevotella. It was found that Coxiella, Prevotella, Subdoligranulum, Akkermansia, and Roseburia were enriched in the 400 mg/kg SRE supplementation group. However, similar changes were not observed in the intestines of fattened pigs fed diets supplemented with 0–800 mg/kg SRE [11]. The microbial community of the pig gut can improve host health by regulating nutrient metabolism, modulating the immune system, and inhibiting intestinal colonization by pathogens [42,43]. Roseburia and Prevotella are two important genera of intestinal cornerstone bacteria that are closely related to intestinal health [44,45]. Some studies have found that the abundance of Roseburia is negatively correlated with the development of several diseases [46,47]. Previous studies have found that the abundance of intestinal butyrate and the growth of pigs are positively correlated with the abundance of Roseburia [48,49,50]. In rats adapted to a fasting situation, metabolism in the liver leads to an increase in serum ketone body levels, but this change was not directly related to changes in colon short-chain fatty acid content [51]. This was because short-chain fatty acids, the main fuel source for the colon epithelium, are mostly absorbed and metabolized [52]. Akkermansia was found to be negatively correlated with inflammatory responses and disorders of fat metabolism in obese mice [53]. In another study, it was noted that Akkermansia exhibited higher abundance in the ileum of pigs with high feed efficiency [54]. Despite the enrichment of intestinal Akkermansia in pigs with diets supplemented with 400 mg/kg SRE in this study, there was no significant improvement in production performance. Intestinal short-chain fatty acids are the main product of intestinal microbial fermentation, and changes in their content are related to changes in intestinal flora [55]. In our study, a significant positive correlation was found between the amount of colon prevotella (genus) and acetic acid content. In addition, our study showed a significant correlation between the number of gut microorganisms and the antioxidant capacity of the body. This result may be related to the activity of intestinal neurons [56]. Enteric neurons can signal to the central nervous system via vagal, endocrine, and immune pathways to maintain the body’s health [57]. Therefore, the relationship between the dynamic balance of intestinal microorganisms and host health needs to be further investigated.

## 5. Conclusions

In summary, the supplementation of the diet with 400 mg/kg SRE can improve piglet health by regulating the redox balance of weaned piglets. This improvement may also be associated with an increase in the abundance of potentially beneficial bacteria, but further research is needed.

## Figures and Tables

**Table 1:** Ingredients and nutrient levels of the basal diet (air-dried basis).

Ingredients	Content, %	Nutrient Level ^(b)^	Content
Corn	28.85	ME, MJ/kg	14.23
Puffed corn	10.00	CP, %	21.07
Puffed soy flour	14.00	Ca, %	1.17
Fermented soybean meal	11.50	STTD P, %	0.37
Soybean meal	7.00	Lys SID, %	1.40
Fish meal	3.00	Met SID, %	0.40
Low-protein whey powder	15.00	Thr SID, %	0.82
Whey protein concentrate	1.00	Trp SID, %	0.23
Soybean oil	1.00		
Sucrose	3.00		
Calcium citrate	1.85		
CaHPO_4_	0.60		
NaCl	0.25		
L-Lys	0.55		
DL-Met	0.15		
L-Thr	0.20		
L-Trp	0.05		
L-Val	0.10		
Choline chloride	0.20		
Premix ^(a)^	1.50		
Total	100.00		

^(a)^ The premix provided the following per kg of diet: VA 4 400IU, VD_3_ 440 IU, VE 30 IU, VK 1 mg, VB_12_ 40 μg, VB_1_ 3 mg, VB_2_ 10 mg, nicotinic acid 40 mg, D-pantothenic acid 15 mg, folic acid 1 mg, VB_6_ 8 mg, biotin 0.08 mg, FeSO_4_•H_2_O 120 mg, CuSO_4_•5H_2_O 16 mg, MnSO_4_•H_2_O 70 mg, ZnSO_4_•H_2_O 120 mg, CaI_2_O_6_ 0.7 mg, Na_2_SeO_3_ 0.48 mg. ^(b)^ Nutrient levels were calculated values, except for the digestible energy, crude protein, and available phosphorus which were determined values.

**Table 2:** Identified components and their contents in stevia residue extract [11] ^(a)^.

Items	Content, %
Chlorogenic acid	13.69
Isochlorogenic acid B	1.62
Isochlorogenic acid C	7.63
Isochlorogenic acid A	16.66
Neochlorogenic acid	2.00
Cryptochlorogenic acid	1.68
Quinic acid	1.30
Caffeic acid	0.30
Ethyl caffeic acid	0.60
Rutin	1.5
Quercetin	0.50
β-glucans	19.50
Xylo-oligosaccharide	1.50
Crude protein	1.70
Crude fat	0.10
Moisture	3.50
Ash	6.50

^(a)^ High-performance liquid chromatography (HPLC) was used for the identification and quantification of isochlorogenic acid A, isochlorogenic acid B, isochlorogenic acid C, chlorogenic acid, neochlorogenic acid, and cryptochlorogenic acid. β-glucan, xylo-oligosaccharide, caffeic acid, ethyl caffeate, rutin, quinic acid, and quercetin were identified using non-targeted high-resolution mass spectrometry and quantified using high-performance liquid chromatography. The contents of moisture, ash, crude protein, and crude fat were determined according to the China National Standard; the document numbers are GB/T 6435-2014, GB/T 6438-2007, GB/T 6432-2018, and GB/T 6433-2006.

**Table 3:** Effects of supplementation with stevia residue extract in the diet on performance and diarrhea rate of weaned piglets ^(a)^.

Items	SRE, mg/kg				*p*-Value
	CON	100	200	400	
**Days 1–28**					
ADG, g	382.62 ± 8.61	393.10 ± 10.36	388.27 ± 13.51	388.21 ± 16.37	0.95
ADFI, g	517.72 ± 5.50	509.33 ± 16.31	495.87 ± 13.47	519.08 ± 20.33	0.68
F/G	1.36 ± 0.04	1.29 ± 0.02	1.28 ± 0.02	1.34 ± 0.03	0.20
Diarrhea rate, %	5.36 ± 1.32	3.57 ± 0.53	3.77 ± 0.66	5.95 ± 1.18	0.27
**Days 29–42**					
ADG, g	562.62 ± 17.85	574.76 ± 13.27	604.29 ± 13.96	573.10 ± 11.65	0.23
ADFI, g	889.91 ± 24.10	932.00 ± 24.96	958.58 ± 16.37	942.08 ± 51.30	0.63
F:G	1.60 ± 0.03	1.62 ± 0.04	1.59 ± 0.04	1.64 ± 0.07	0.88
Diarrhea rate, %	13.69 ± 1.89 ^a^	6.35 ± 1.94 ^b^	3.37 ± 0.37 ^b^	7.74 ± 1.30 ^ab^	<0.01
**Days 1–42**					
ADG, g	442.62 ± 8.03	453.65 ± 8.44	460.28 ± 9.43	449.84 ± 14.04	0.68
ADFI, g	645.12 ± 9.21	650.22 ± 17.83	650.11 ± 12.47	660.08 ± 29.34	0.95
F/G	1.46 ± 0.02	1.43 ± 0.02	1.41 ± 0.02	1.46 ± 0.03	0.33
Diarrhea rate, %	8.13 ± 1.37 ^a^	4.50 ± 0.63 ^ab^	3.64 ± 0.50 ^b^	6.55 ± 0.97 ^ab^	0.01

^(a)^ Values are means ± SEM, n = 6. Within a row, means without a common superscript letter differ at *p* < 0.05. SRE: stevia residue extract. Con: control group without the supplementation of stevia residue extract in the diet; 100: diets supplemented with 100 mg/kg stevia residue extract group; 200: diets supplemented with 200 mg/kg stevia residue extract group; 400: diets supplemented with 400 mg/kg stevia residue extract group. ADG: average daily gain; ADFI: average daily feed intake; F:G: feed-to-gain ratio.

**Table 4:** Effects of supplementation with stevia residue extract in the diet on the serum antioxidant capacity of weaned piglets on days 28 and 42 of the trial ^(a)^.

Items	SRE, mg/kg				*p*-Value
	con	100	200	400	
**Day 28**					
MDA, nM/mL	3.79 ± 0.23 ^a^	1.78 ± 0.16 ^b^	2.10 ± 0.03 ^b^	2.19 ± 0.06 ^ab^	<0.01
T-AOC, mM/L	0.32 ± 0.02 ^b^	0.38 ± 0.02 ^ab^	0.40 ± 0.01 ^a^	0.43 ± 0.02 ^a^	0.02
T-SOD, U/mL	112.06 ± 7.53 ^c^	130.37 ± 5.97 ^bc^	167.30 ± 4.53 ^a^	148.04 ± 2.60 ^abc^	<0.01
CAT, U/mL	4.54 ± 0.14	4.92 ± 0.56	5.28 ± 0.60	4.70 ± 0.13	0.88
GSH-PX, U/mL	415.28 ± 22.47 ^b^	455.06 ± 7.08 ^ab^	460.73 ± 10.96 ^ab^	486.57 ± 15.49 ^a^	0.03
**Day 42**					
MDA, nM/mL	2.90 ± 0.21 ^a^	1.70 ± 0.09 ^b^	2.07 ± 0.10 ^b^	1.97 ± 0.24 ^b^	0.01
T-AOC, mM/L	0.30 ± 0.01	0.30 ± 0.01	0.28 ± 0.02	0.26 ± 0.02	0.365
T-SOD, U/mL	185.26 ± 4.32 ^b^	220.86 ± 5.37 ^a^	226.86 ± 7.64 ^a^	228.72 ± 7.84 ^a^	<0.01
CAT, U/mL	2.64 ± 0.16 ^b^	6.81 ± 0.50 ^a^	4.66 ± 0.27 ^ab^	4.79 ± 0.94 ^ab^	<0.01
GSH-PX, U/mL	601.32 ± 27.39 ^b^	610.53 ± 39.31 ^b^	847.81 ± 26.78 ^a^	972.77 ± 51.20 ^a^	<0.01

^(a)^ Values are means ± SEM, n = 6. Within a row, means without a common superscript letter differ at *p* < 0.05. SRE: stevia residue extract. Con: control group without the supplementation of stevia residue extract in the diet; 100: diets supplemented with 100 mg/kg stevia residue extract group; 200: diets supplemented with 200 mg/kg stevia residue extract group; 400: diets supplemented with 400 mg/kg stevia residue extract group. MDA: malondialdehyde; T-AOC: malondialdehyde; T-SOD: total superoxide dismutase; CAT: catalase; GSH-Px: glutathione peroxidase.

**Table 5:** Effects of supplementation with stevia residue extract in the diet on the liver antioxidant capacity of weaned piglets on day 42 of the trial ^(a)^.

Items	SRE, mg/kg				*p*-Value
	CON	100	200	400	
MDA, nM/mg prot	0.54 ± 0.09 ^a^	0.39 ± 0.05 ^ab^	0.27 ± 0.01 ^b^	0.32 ± 0.02 ^ab^	<0.01
T-AOC, mM/g prot	0.67 ± 0.04	0.68 ± 0.03	0.63 ± 0.03	0.70 ± 0.04	0.58
T-SOD, U/mg prot	119.43 ± 3.09 ^ab^	128.79 ± 4.71 ^a^	109.43 ± 4.03 ^b^	106.57 ± 2.65 ^b^	0.02
CAT, U/mg prot	349.64 ± 9.06 ^b^	291.05 ± 23.20 ^b^	321.036 ± 18.01 ^b^	480.78 ± 10.14 ^a^	<0.01
GSH-PX, U/mg prot	161.99 ± 8.66 ^b^	166.72 ± 6.77 ^ab^	159.93 ± 3.96 ^b^	191.00 ± 5.77 ^a^	0.01

^(a)^ Values are means ± SEM, n = 6. Within a row, means without a common superscript letter differ at *p* < 0.05. SRE: stevia residue extract. Con: control group without the supplementation of stevia residue extract in the diet; 100: diets supplemented with 100 mg/kg stevia residue extract group; 200: diets supplemented with 200 mg/kg stevia residue extract group; 400: diets supplemented with 400 mg/kg stevia residue extract group. MDA: malondialdehyde; T-AOC: malondialdehyde; T-SOD: total superoxide dismutase; CAT: catalase; GSH-Px: glutathione peroxidase.

**Table 6:** Effects of supplementation with stevia residue extract in the diet on the small intestinal enzyme activity of weaned piglets on day 42 of the trial ^(a)^.

Items	SRE, mg/kg				*p*-Value
	CON	100	200	400	
**Duodenum**					
Trypsin, U/mg port	9.05 ± 0.86	5.75 ± 0.84	6.01 ± 0.88	7.72 ± 0.98	0.07
Amylase, U/mg port	0.64 ± 0.08 ^a^	0.45 ± 0.04a ^b^	0.36 ± 0.06 ^b^	0.35 ± 0.10 ^b^	0.05
Lipase, U/g port	6539.95 ± 1095.13	4729.14 ± 563.82	4051.90 ± 404.88	6017.10 ± 456.59	0.07
**Jejunum**					
Trypsin, U/mg port	8.52 ± 0.63	6.93 ± 0.57	7.80 ± 1.04	6.37 ± 0.79	0.34
Amylase, U/mg port	0.52 ± 0.10	0.45 ± 0.11	0.40 ± 0.09	0.49 ± 0.10	0.85
Lipase, U/g port	4077.81 ± 556.84	2894.77 ± 541.50	3014.59 ± 1000.54	4523.14 ± 815.02	0.36
**Ileum**					
Trypsin, U/mg port	14.27 ± 1.40	13.78 ± 1.63	13.82 ± 0.86	11.26 ± 0.28	0.28
Amylase, U/mg port	0.53 ± 0.11	0.36 ± 0.09	0.25 ± 0.08	0.36 ± 0.11	0.30
Lipase, U/g port	8291.51 ± 578.83	6620.19 ± 1260.78	6310.32 ± 1079.86	7051.24 ± 1171.35	0.58

^(a)^ Values are means ± SEM, n = 6. Within a row, means without a common superscript letter differ at *p* < 0.05. SRE: stevia residue extract. Con: control group without the supplementation of stevia residue extract in the diet; 100: diets supplemented with 100 mg/kg stevia residue extract group; 200: diets supplemented with 200 mg/kg stevia residue extract group; 400: diets supplemented with 400 mg/kg stevia residue extract group.

**Table 7:** Effects of supplementation with stevia residue extract in the diet on the content of short-chain fatty acids in the colonic contents of weaned piglets on day 42 of the trial ^(a)^.

Items	SRE, mg/kg				*p*-Value
	CON	100	200	400	
Acetic acid, nmol/g	8.41 ± 0.50	9.49 ± 0.46	9.56 ± 0.54	9.75 ± 0.45	0.23
Propionic acid, nmol/g	6.72 ± 0.68	6.54 ± 0.31	6.55 ± 0.60	6.48 ± 0.34	0.99
Isobutyric acid, nmol/g	0.96 ± 0.17 ^a^	0.49 ± 0.04 ^b^	0.69 ± 0.13 ^ab^	0.78 ± 0.06 ^a^	0.03
Butyric acid, nmol/g	2.08 ± 0.19	2.01 ± 0.22	2.03 ± 0.23	2.36 ± 0.30	0.70
Isovaleric acid, nmol/g	1.05 ± 0.23 ^a^	0.41 ± 0.07 ^b^	0.68 ± 0.17 ^ab^	0.76 ± 0.08 ^a^	0.04
Valeric acid, nmol/g	0.91 ± 0.13	0.66 ± 0.15	0.74 ± 0.11	1.02 ± 0.17	0.283

^(a)^ Values are means ± SEM, n = 6. Within a row, means without a common superscript letter differ at *p* < 0.05. SRE: stevia residue extract. Con: control group without the supplementation of stevia residue extract in the diet; 100: diets supplemented with 100 mg/kg stevia residue extract group; 200: diets supplemented with 200 mg/kg stevia residue extract group; 400: diets supplemented with 400 mg/kg stevia residue extract group.

## Data Availability

The data are contained within the article. The results of 16s rRNA sequencing can be found at: https://www.ncbi.nlm.nih.gov/bioproject/?term=PRJNA881625 (accessed on 24 September 2022).
